# Time Lag between COVID-19 Diagnosis and Symptoms Onset for Different Population Groups: Evidence That Self-Testing in Schools Was Associated with Timely Diagnosis among Children

**DOI:** 10.3390/life12091305

**Published:** 2022-08-25

**Authors:** Kassiani Mellou, Spyros Sapounas, Ioannis Panagoulias, Maria Gkova, Kalliopi Papadima, Anastasia Andreopoulou, Danai Kalotychou, Michalis Chatzopoulos, Kassiani Gkolfinopoulou, Vassiliki Papaevangelou, Sotirios Tsiodras, Georgios Panagiotakopoulos, Theoklis Zaoutis, Dimitrios Paraskevis

**Affiliations:** 1 National Public Health Organization, 15123 Athens, Greece; 2 Third Department of Pediatrics, University Hospital Attikon, National and Kapodistrian University of Athens, 12462 Athens, Greece; 3 34th Department of Internal Medicine, Attikon University Hospital, Medical School, National and Kapodistrian University of Athens, 11527 Athens, Greece

**Keywords:** COVID-19, SARS-CoV-2, testing, self-test, diagnosis

## Abstract

Early identification of COVID-19 cases has been vital for reducing transmission and enabling treatment. In Greece, in autumn 2021 when Delta was the predominant circulating variant, unvaccinated citizens had to be tested before attending activities, and self-testing was required twice a week for students (5–17 years). Here, we describe the time of diagnosis by age group and possible exposure to assess testing strategies (September to November 2021). Information on the presence of symptoms at the time of diagnosis was available for 69,298 cases; 24,855 (36%) were asymptomatic or tested the same day as onset (early diagnosis), 21,310 (31%) reported testing one day after, and 23,133 (33%) did so two or more days after the onset of symptoms. The median lag was 2 days (1–14). Early diagnosis significantly differed among age groups (*p*-value < 0.001) and was higher among children. For every one-year increase of age, the odds of an early diagnosis were reduced by 1%. Cases exposed during training activities or in settings such as accommodation centers and hospitals were more frequently diagnosed early. The percentage of persons having a positive self-test before a rapid test/PCR diagnosis ranged from 7% in the age group of 60 years and above to 86% in the age group of 5–17 years. The provision of self-tests in schools and increased testing in closed settings led to an earlier diagnosis and probably to a decreased transmission of the virus in the period during which Delta was the predominant variant in Greece. However, more effort is needed for early diagnosis of adults in the community, especially after the onset of symptoms.

## 1. Introduction

On 26 February 2020, the first case of coronavirus disease 2019 (COVID-19) was detected in Greece [1]. The risk of exceeding healthcare systems’ capacity has led to the implementation of measures, such as distancing and wearing masks in public places.

It very soon became apparent that an early identification of cases was vital for reducing the further transmission of the virus, enabling a rapid initiation of isolation, contact tracing and treatment [2,3]. The importance of early testing had been known from the management of previous MERS and SARS outbreaks [4,5]. Evidence that SARS-CoV-2 is also commonly transmitted from asymptomatic persons further increased the need for making testing widely available [6,7].

During the first months of the pandemic, the laboratory capacity for PCR testing was limited. Respiratory specimens, both nasopharyngeal swabs or endotracheal aspirates, were collected mainly from symptomatic cases in medical services, and few laboratories were accredited at the time for COVID-19 testing [8].

Although more laboratories were gradually accredited, a wide accessibility to PCR testing was not possible, as these tests are expensive, equipment-based, time-consuming and requiring biological expertise.

As rapid antigen tests became widely available for the detection of the virus and self-tests were released in the public health market, authorities emphasized the need for frequent testing that would allow the early detection of cases [9,10,11,12,13].

In Greece, the substantial scale-up of the testing capacity led to a series of measures for the protection of the population. Specifically, since the opening of schools for the new academic year 2021–2022 on 14 September 2021, self-testing was required twice a week for students 5–17 years of age, and results had to be reported on an online platform. Based on the protocol for the management of COVID-19 cases, close contacts of cases in schools were also tested with rapid tests. Rapid tests were also requested from unvaccinated citizens in order for them to attend their work or join social activities. Routine testing (usually once per week) was also performed in accommodation centers for migrants and refugees, hospitals, prisons and other closed facilities [14,15].

From September to November 2021, as Delta swept rapidly through the country and became the predominant variant the recorded number of cases and fatalities in Greece substantially increased.

The aim of this study is to describe testing practices among the recorded COVID-19 cases in the aforementioned period and to identify difference in time of diagnosis by age group, vaccination status, geographical region of the country and possible exposure to the virus, so as to assess the testing guidance for the population and take measures for the enhancement of early diagnosis in the future, especially as the transmissibility of the virus increases.

## 2. Method

### 2.1. Data Sources

The Greek National Public Health Organization (EODY) is, by law, the responsible entity in the country for the epidemiological surveillance and response. All diagnosed COVID-19 cases (symptomatic and asymptomatic) are recorded on the Hellenic National COVID-19 registry, which was designed specifically for the management of the pandemic.

Since 28 November 2020 (date of sample collection), cases with a positive rapid test started to be recorded at the National COVID-19 registry without the need for a PCR confirmation to be a prerequisite.

Data on COVID-19 cases during the study period (September–November 2021) were retrieved from the registry.

Vaccination coverage data by regional units (Nomenclature of Territorial Units for Statistics—NUTS-3) of the country were retrieved by the national vaccine tracker of the Ministry of Health.

### 2.2. Study Population

Regional units of islands were excluded from the analysis due to the substantial fluctuations of the population during touristic periods and to different vaccination programs implemented during the summer. The four regional units of Athens were analyzed as one. Overall, 41 different regional units were included in the analysis (mainland), accounting for 87% of the Greek population. Sixty-six cases that tested positive more than 14 days after the onset of symptoms were excluded from the analysis.

### 2.3. Statistical Analysis

The time lag between the date of testing (PCR or rapid test) and the date of onset of symptoms (for symptomatic cases) was calculated. Three case categories were defined: (i) early diagnosed cases as those tested positive in the absence of symptoms or the same day they developed symptoms, (ii) cases diagnosed one day after the onset of symptoms, and (iii) cases diagnosed two or more days after the onset of symptoms.

The proportion of cases for each of the aforementioned categories was estimated by ISO (International Organization for Standardization) week, age group, sex, regional unit of the country and most probable exposure (family contact, social outdoor event, educational activity, workplace, social indoor event, market shop, transportation, restaurants, healthcare facilities, nursing homes, accommodation facilities for refugees/migrants, military camps, prisons).

The age categories used were 0–4, 5–9, 10–14, 15–17, 18–24, 25–49, 50–59, 60–69, 70–79 and 80+ years.

The proportion of reported cases having a positive self-test before the rapid test/PCR diagnosis was also calculated by time of diagnosis over time.

The seven-days moving averages of cases overall and of cases having a positive self-test before the rapid test/PCR diagnosis were plotted, as there was a fluctuation of the number of recorded cases by the day of the week.

The daily proportions of early diagnoses were assessed using the augmented Dickey-Fuller (ADF) test. The same test was used for the assessment of the daily proportion of cases diagnosed with self-tests before the positive PCR/rapid test.

An association was considered statistically significant when *p* ≤ 0.05; all statistical tests were two-tailed.

The statistical analysis was conducted using Stata 16 statistical software (StataCorp. Stata Statistical Software: Release 16; StataCorp LLC: College Station, TX, USA, 2019 [16].

## 3. Ethics Approval

EODY is authorized by Greek law to process COVID-19 epidemiological data for public health purposes. No personal data of the recorded cases were used. Data was managed in accordance with the national and European Union regulations.

## 4. Results

Information on the presence of symptoms at the time of diagnosis was available for 69,298 COVID-19 cases for the time period between 14 September and 30 November. In total, 24,855 (36%) were asymptomatic at the time of the diagnosis or took a test the same day as the onset of symptoms (early diagnosis), 21,310 (31%) reported testing one day after the onset of symptoms, and 23,133 (33%) did so two or more days after the onset of symptoms. The median time from the onset of symptoms to diagnosis was two days (range 1–14) for cases that were symptomatic at the time of testing. Most cases (90%) reported a diagnosis up to five days after the onset of symptoms.

**Figure 1:**
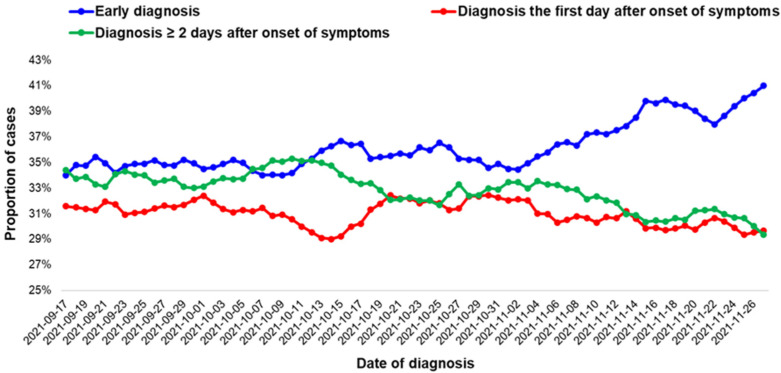
Seven-days moving averages of COVID-19 cases that tested positive in the absence of symptoms or at the first day of the onset of symptoms (early diagnosed cases), for COVID-19 cases diagnosed the first day after onset of symptoms and for COVID-19 cases diagnosed two or more days after the onset of symptoms, by date of diagnosis, Greece, 17 September–27 November 2021.

The moving average of the proportion of early diagnosed cases seems to be increasing; however, no statistically significant trend was found (Figure 1).

### 4.1. Time of Diagnosis, Sex and Age

The proportion of early diagnoses was 38% among males and 34% among females.

**Figure 2:**
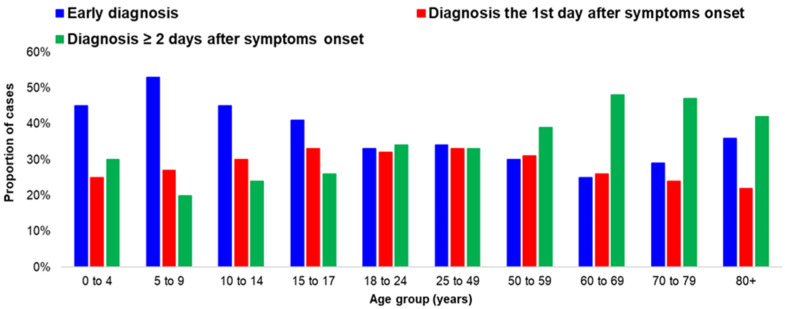
Proportion of COVID-19 infection cases by time of diagnosis (before onset of symptoms or on the same day, one day after the onset of symptoms and two days or more after the onset of symptoms) by age group; Greece, September–November 2021.

The proportion of early diagnosed cases significantly differed among age groups (*p*-value < 0.001). A higher proportion was recorded in children 0–17 years of age. Half of the children aged 5–9 years were diagnosed early, while the respective proportion was less than 30% for ages 50–79 years (Figure 2). Age had a statistically significant association with the proportion of early diagnoses (*p*-value < 0.001). Increased age was associated with a decreased proportion of early diagnoses (For every one-year increase of age, the odds of an early diagnosis was reduced by 1%.). The proportion of early diagnosed cases increased for cases aged 80 years or more.

Among the symptomatic cases at the time of diagnosis, the median time between the onset of symptoms and diagnosis was one day (range: 1–14) for cases less than 50 years of age and two days (range: 1–14) for cases equal or more than 50 years. The lag between the onset of symptoms and diagnosis was higher in older ages (*p*-value: < 0.001).

### 4.2. Time of Diagnosis by Regional Unit of the Country

**Figure 3:**
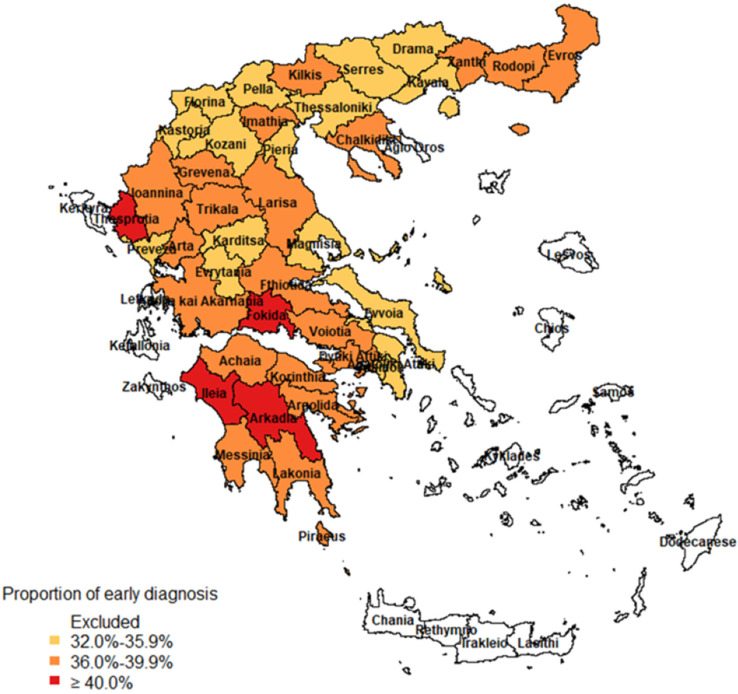
Proportion of COVID-19 cases that tested positive in the absence of symptoms or on the first day of the onset of symptoms (early diagnosed cases), by regional unit; Greece, September–November 2021.

The median proportion of early diagnoses by regional unit was 37% (range: 32.1–49.5%) (Figure 3). In 21 of the 41 regional units of the study, 36.0–39.9% of the cases were detected early, while in 16 of them, the respective proportion was 32.0–35.9%. In four regional units, more than 40.0–50.0% of the cases were detected before the onset of the symptoms or at the same day as the symptoms started.

### 4.3. Time of Diagnosis and Possible Exposure Category

**Figure 4:**
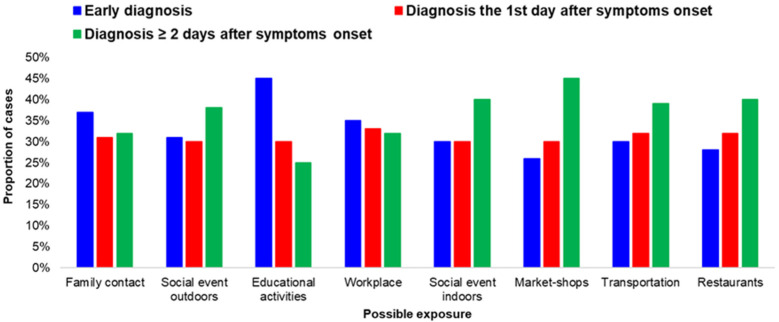
Proportion of early diagnosed cases of COVID-19 infection (diagnosis before onset of symptoms or on the first day of the onset of symptoms), by potential family exposure or exposure in community settings; Greece, September–November 2021.

The proportion of early diagnoses differed by possible exposure category (Figure 4).

**Figure 5:**
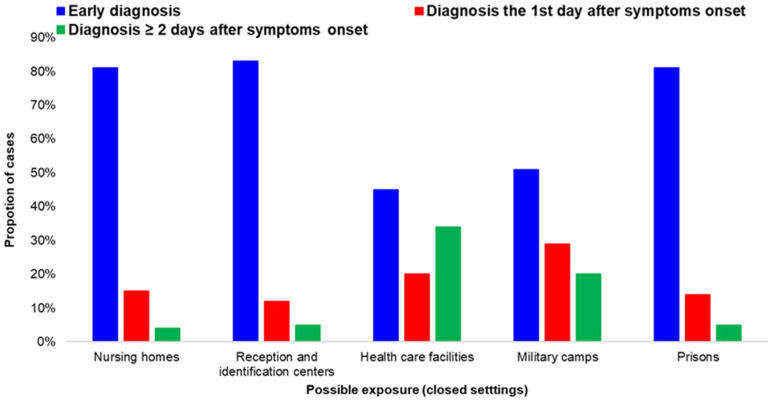
Proportion of early diagnosed cases of COVID-19 infection (diagnosis before onset of symptoms or on the first day of the onset of symptoms), by potential exposure category in closed settings and healthcare facilities; Greece, September–November 2021.

Cases exposed during training activities (at schools, educational programs, etc.) had a high percentage of early diagnoses. The same applied for closed settings, such as prisons and accommodation centers, as well as among people exposed at hospitals and other healthcare facilities (Figure 5). 

In the age group of 80 years or more, 135 of the 567 (24%) cases with early diagnosis were residents of nursing homes or reported a possible exposure at healthcare services, while the respective percentage among cases 70–79 years of age was 8%.

A lower rate of early diagnosis was recorded among cases that considered social activities, such as eating outside or visiting shops, to be their most probable exposure to the virus.

### 4.4. Time of Diagnosis and Vaccination Coverage by Regional Unit

**Figure 6:**
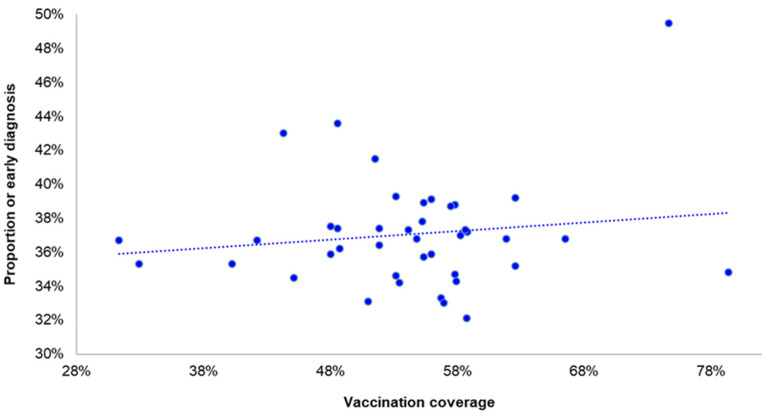
Proportion of early diagnosed COVID-19 infections (diagnosis before onset of symptoms or on the first day of the onset of symptoms) and vaccination coverage of the population by regional unit; Greece, September–November 2021.

As shown in Figure 6, there was not a statistically significant association of the early diagnosis with the vaccination coverage by regional unit (*p* = 0.45).

The same applies for children and adolescents. The early diagnosis of COVID-19 infection among children of 5–17 years was not associated with the vaccination coverage of the population by regional units of the country (*p* = 0.24).

### 4.5. Contribution of Self-Tests

Of the 69,298 recorded cases, 22,235 (32%) had a positive self-test before the rapid test/PCR diagnosis: 39% of the cases in the age group 0–4, 86% in the age group 5–17, 15% in the age group 18–59 and 7% in the age group 60 years and above.

**Figure 7:**
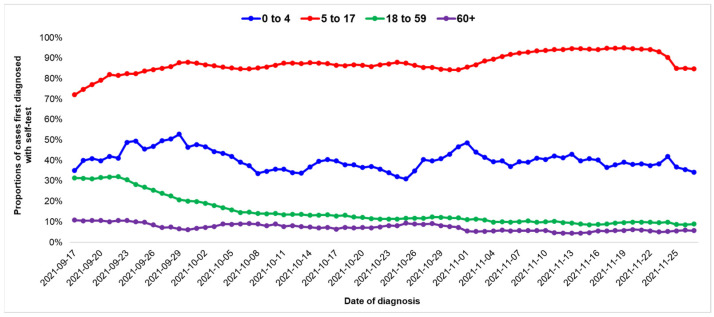
Moving average of proportions of cases of COVID-19 infection with a positive self-test prior to the PCR/rapid test diagnosis for age groups 0–4, 5–17, 18–59 and 60 years and older, by date of diagnosis; Greece, September–November 2021.

The proportion of cases with a positive self-test prior to the PCR/rapid test diagnosis decreased over time solely in the age group of 18–59 years (*p*-value at ADF test = 0.58) (Figure 7).

There were no significant differences by geographical region. The median proportion of cases with a positive self-test was 33% (24–39%).

**Figure 8:**
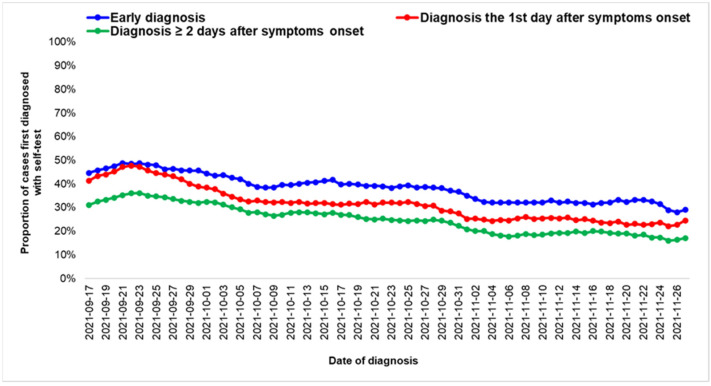
Moving average of proportions of cases of COVID-19 infection with a positive self-test prior to the PCR/rapid test diagnosis for COVID-19 cases that tested positive in the absence of symptoms or on the first day of the onset of symptoms (early diagnosed cases), for COVID-19 cases diagnosed the first day after the onset of symptoms and for COVID-19 cases diagnosed two or more days after the onset of symptoms; Greece, September–November 2021.

When conducting an analysis by time of diagnosis, 9486 of the 24,855 (38%) early diagnosed cases had a positive self-test prior to the rapid test/PCR diagnosis, with the respective percentage being 32% (6843 of the 21,310) and 26% (5906 of the 23,133) among cases that were diagnosed one day and two or more days after the onset of symptoms (Figure 8).

## 5. Discussion

COVID-19 is highly transmissible and rapidly spread, as it can be transmitted by respiratory droplets and contact [17]. The long incubation period and non-existent-to-moderate symptoms make the identification, tracing, and elimination of coronavirus COVID-19 disease unexpectedly difficult [18].

Early diagnosis is key to surveillance and outbreak management and should be used to inform infection prevention measures [3]. The detection of cases at an early stage is needed to immediately isolate infected people from healthy ones. Thus, diagnostic testing in high volumes and the rapid availability of results have become important tools to prevent the further spread of the disease [19]. By analyzing the time of diagnosis in relation to the onset of symptoms, we were able to assess how these public health actions increased early diagnosis.

Overall, due to the high testing capacity and substantial access of the population to testing, the proportion of early diagnosed cases is high. The proportion of early diagnosis was higher among children of 5–17 years, showing that the introduction of a self-testing strategy for the school population was successful. There is evidence that the provision of home self-tests has improved the testing perception of the population and that self-testing can provide similar results to professional testing [20]. As it is widely acceptable that we need to keep schools open, there is an urgent need to implement effective strategies for the early detection of the infection and prevention of COVID-19 transmission [21]. This strategy will also promote the control of COVID-19 in the community. However, fewer cases were proportionally recognized in a timely way among older people. This is crucial, as increased age is a well-known risk factor for the development of severe disease. Possibly, people older than 50 years old should also be advised to routinely self-test and avoid delays in testing when they experience symptoms, even mild ones. The decision of the Greek government to provide self-tests free of charge for everyone during the Christmas holidays was in accordance with the results of this study and is considered to have increased the early diagnosis of cases, especially in the non-working older population of the country.

The finding that there are still people diagnosed 2–14 days after the development of their first symptoms shows that efforts to increase awareness and testing are still needed.

A higher proportion of elderly adults, older than 80 years, were diagnosed in a timely way, most likely because almost one third of them either represent residents of nursing homes or reported possible exposure at healthcare services. In such cases, testing practices differ compared to the community.

A high proportion of early diagnosis among cases exposed during training activities (at schools, educational programs, etc.) possibly displays the effects of increased testing in the school environment.

Similarly, the percentage of early diagnosis was high among people residing in nursing homes and long-term care facilities where routine testing has been implemented for both the personnel and hosted population and where testing of contacts has been exhaustive. Evidence showed that frequent testing and rapid turnaround times of test results yielded a higher probability of early detection of infections, and hence the prevention of outbreaks, in such at-risk settings [22,23]. This finding is important, as long-term care facilities (LTCFs) have been a conspicuous source of COVID-19 morbidity and mortality throughout the pandemic [24,25,26].

Data regarding the time of diagnosis in accommodation centers for refugees and migrants also indicated that the strategy of routine testing of the hosted population and the personnel has helped in the containment of clusters in the hosting facilities and in the early implementation of measures. It has been previously documented that in the pre-vaccination period, 48% of the cases diagnosed in hosting camps and reception and identification centers were asymptomatic, with the respective percentage in the general population being 80% (*p* < 0.001) [27]. The early detection of cases and the prevention of transmission in the susceptible population living in such facilities has been a priority in the country, as overcrowding and frequent close contact among residents (e.g., food lines, shared toilets) could easily lead to the rapid spread of COVID-19 [28].

On the other hand, in the community, routine testing was not possible, and thus, lower percentages of early diagnoses among cases exposed in social activities were recorded.

The lack of geographical differences in the time of testing is a finding that supports the fact that the laboratory capacity is similar across the mainland and that access to testing is wide and not impaired by geographical obstacles.

The finding that late diagnosis and vaccination hesitancy in the population were not correlated suggests that everyone has access to testing and that hesitancy towards the COVID-19 vaccine is not accompanied, at least significantly, with hesitancy against testing.

Even though early diagnosis increased during the study period, the proportion of cases that had a positive self-test before rapid test/PCR decreased among adults 18-59 years of age, probably due to testing fatigue. The benefit of using self-tests should be stressed in all age groups.

One main limitation of the study was that the data focused on investigated cases only. Cases that did not provide a valid telephone number at the medical service or refused to answer questions from public health professionals on their symptoms and possible exposures were not included in the study. This may have introduced a bias, since such cases probably differed from the responders, and the results may have therefore not been representative of the whole population. Cases that were reluctant to give information to public health services might also have been hesitant to visit healthcare services and get tested.

Additionally, since possible exposure is based on the assumed self-reported exposure revealed during an investigation, this information cannot be validated, especially in the community.

Finally, the category of late diagnosed cases (two or more days after the onset of symptoms) is heterogeneous; however, the analysis of data did not identify significant differences within this group. Moreover, most cases were diagnosed within a period of five days after symptoms’ onset. In the future, it would be interesting to further investigate the reasons that lead to some cases being diagnosed after several days of symptoms.

## 6. Conclusions

Based on the results presented here, some strategies that aimed at the early detection of COVID-19 infections in the country were effective and probably led to a decreased transmission of the virus in the period during which Delta was the predominant variant in Greece.

The provision of self-tests and campaigns for promoting early testing has led to a high number of early diagnosed cases and a subsequent early implementation of measures, especially in school settings. Additionally, continuous, and sustained testing in closed populations such as healthcare facilities and prisons as well as extensive testing in hospitals led to early diagnosis among these populations. As a consequence of this, larger or prolonged outbreaks were prevented in such settings. Moreover, subsequent transmissions in the community were also controlled due to the effective testing in schools, close settings, and healthcare facilities.

In our times and given the availability of antivirals, an effective strategy for an early diagnosis of older people is needed, especially after the emergence of symptoms. As the pandemic continues to spread, a continuous effort will be needed to address the anticipated testing fatigue of the population and to increase early diagnosis, especially among populations at a high risk for severe disease.

## Data Availability

Data were extracted by the National COVID-19 Registry.
